# Internet-of-Things-Based Multiple-Sensor Monitoring System for Soil Information Diagnosis Using a Smartphone

**DOI:** 10.3390/mi14071395

**Published:** 2023-07-08

**Authors:** Yin Wu, Zenan Yang, Yanyi Liu

**Affiliations:** College of Information Science and Technology, Nanjing Forestry University, Nanjing 210037, Chinayyliu@njfu.edu.cn (Y.L.)

**Keywords:** soil information, wireless sensor, mobile phone app, deep Q network, smart agriculture

## Abstract

The rise of Internet of Things (IoT) technology has moved the digital world in a new direction and is considered the third wave of the information industry. To meet the current growing demand for food, the agricultural industry should adopt updated technologies and smart agriculture based on the IoT which will strongly enable farmers to reduce waste and increase productivity. This research presents a novel system for the application of IoT technology in agricultural soil measurements, which consists of multiple sensors (temperature and moisture), a micro-processor, a microcomputer, a cloud platform, and a mobile phone application. The wireless sensors can collect and transmit soil information in real time with a high speed, while the mobile phone app uses the cloud platform as a monitoring center. A low power consumption is specified in the hardware and software, and a modular power supply and time-saving algorithm are adopted to improve the energy effectiveness of the nodes. Meanwhile, a novel soil information prediction strategy was explored based on the deep Q network (DQN) reinforcement learning algorithm. Following the weighted combination of a bidirectional long short-term memory, online sequential extreme learning machine, and parallel extreme machine learning, the DQN Bi-OS-P prediction model was obtained. The proposed data acquisition system achieved a long-term stable and reliable collection of time-series soil data with equal intervals and provided an accurate dataset for the precise diagnosis of soil information. The RMSE, MAE, and MAPE of the DQN Bi-OS-P were all reduced, and the R2 was improved by 0.1% when compared to other methods. This research successfully implemented the smart soil system and experimentally showed that the time error between the value displayed on the mobile phone app and its exact acquisition moment was no more than 3 s, proving that mobile applications can be effectively used for the real-time monitoring of soil quality and conditions in wireless multi-sensing based on the Internet of Things.

## 1. Introduction

Precision agriculture, also known as precision farming or fine farming, originated in the United States in the 1980s. Precision farming is a new type of agriculture supported by information technology and represents a complete set of modern farming operations and management systems that can be positioned, timed, and quantified according to spatial variation [1]. However, at present, the scale of precision agriculture globally is quite limited, and the vast majority of countries still rely on traditional manual farming and human experience for management, which wastes large amounts of human and material resources and generally creates problems such as high costs and low efficiency. These techniques are also no longer applicable to the urgent needs of the current developments in modern agriculture. At the same time, the rapid development of sensors and the Internet of Things (IoT) [2] has brought new development opportunities to the farming industry. The application of IoT technology in farm production practices to gain timely access to production information is of great significance in changing present agricultural processes and ensuring high crop yields and green health [3,4].

In order to meet these growing demands, IoT-based smart agriculture must be intensively studied. This will enable growers and farmers to reduce waste and increase productivity through a variety of methods, from optimizing fertilizer use to improving the efficiency of farm vehicle routes. Smart farming is the application of smart sensors and software to control agricultural production through mobile or computer platforms, making traditional farming more intelligent [5]. In general, the role of the IoT is to connect agricultural animals or plants, agricultural equipment, and agricultural facilities to a network through wireless or wired communications, so that every animal and plant can be managed accurately to achieve the best possible yield and minimize costs [6]. In recent years, IoT technology has been applied to many areas of agriculture, including agricultural environmental monitoring, greenhouse gas emission control, water-saving irrigation, weather monitoring, product safety and traceability, and intelligent equipment diagnosis and management [7,8,9]. Its main goal is to enable the combination of historical analyzed data and real-time inspection data to provide a more accurate model and optimization solution, with the ultimate aim of achieving sustainability in future intelligent agriculture.

Soil is one of the environmental factors that cannot be ignored in the cultivation of crops. As an important medium for the survival of crop roots, the soil tillage layer contains the nutrients and water needed for crop growth. The suitability of the soil environment for the growth of crop roots is of great importance for high-quality and efficient crop cultivation. Thus, more attention and research are needed regarding the soil layer, especially its moisture content (MC) and temperature. High-quality time-series predictions of soil MC and temperature in the tillage layer are significant for both scientific study and practical agricultural production [10].

**Figure 1:**
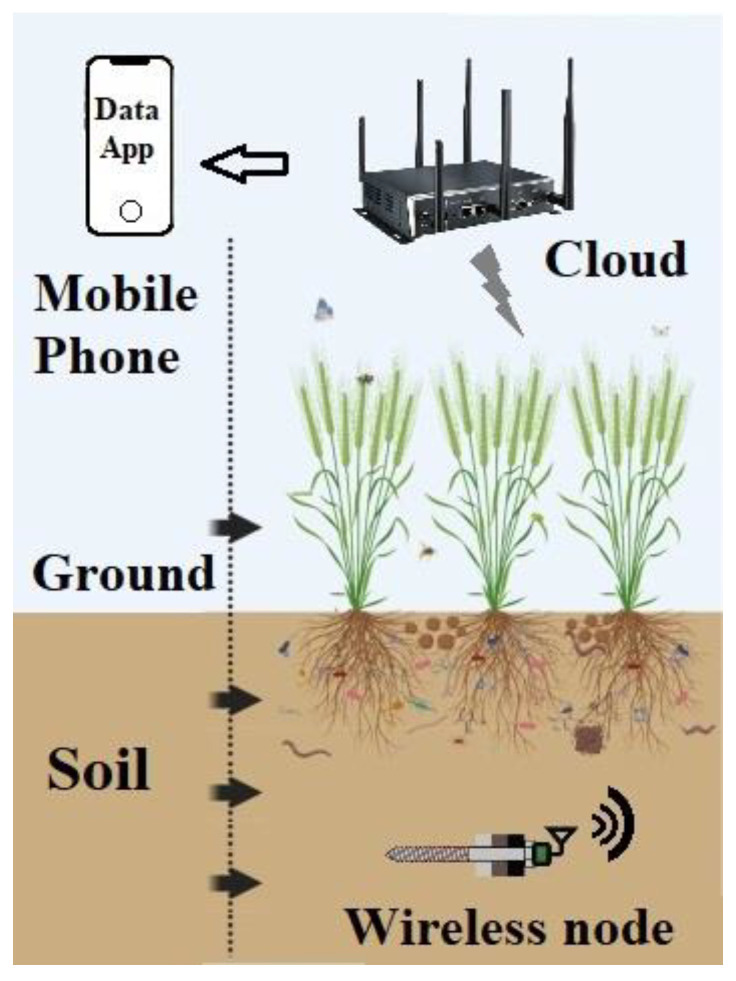
Soil information diagnosis system architecture.

In terms of the smart soil monitoring process, the IoT is divided into three parts, including the sensing device part, the communication part, and the intelligent processing part, which involves using various intelligent technologies to sense and transmit data. Simultaneously, the information is analyzed and processed to achieve optimal monitoring and control, as shown in Figure 1. Our contributions are briefly described as follows:

(1) A complete wireless measurement system used to measure the soil temperature and MC was constructed, and the detailed indicators can be viewed in real time on a mobile phone application.
(2) A deep Q network (DQN)-based soil temperature and MC prediction method was proposed in order to make an informed decision in cases where uncontrolled variations in the soil properties occur.
(3) A measuring campaign was carried out on a farm for 12 months, and the multi-layer soil temperature and MC were recorded and analyzed; the results show that the soil properties can be predicted accurately and efficiently.

Many researchers have now developed a variety of agricultural information systems to meet the needs of different developments. Sun Yanjing et al. described an overall framework for the implementation of agricultural information systems [11]; the overall system consists of a three-layer network: a wireless sensor network at the bottom, a GSM/GPRS/GPS network in the middle, and an internet network at the top. The bottom and middle layers are connected by gateway nodes, while the middle and top layers are connected by public telecommunication gateways. Jirapond Muangprathub et al. designed and developed a control system using node sensors in the crop field with data management via a smartphone and a web application; the system can send notifications through LINE API on the LINE application, and the results showed that this method was useful in agricultural applications [12]. Shadi Atalla et al. proposed a new classification method for the agricultural IoT based on multiple factors and introduced performance evaluation indicators for fixed and mobile scenarios in 6LowPAN networks for precision agriculture [13]. Huan Juan et al. developed a narrow band (NB) IoT-based water quality monitoring system for aquaculture ponds, using a cloud platform for data monitoring. The system uses an STM32L151C8 microcontroller and sensor terminals to collect water quality data such as temperature, pH, dissolved oxygen, etc., in real time and to achieve data aggregation and long-distance transmission to the IoT telecom cloud platform [14]. Shola Usharani et al. designed a system that uses multiple sensors and IoT hardware simultaneously to remotely monitor agricultural fields, efficiently and harmlessly dealing with the problem of animals invading fields [15].

As for smart soil data collection, Yang Weizhong et al. developed an NB IoT-based soil moisture monitoring system which realizes the real-time monitoring of soil moisture [16]. The system probe has a circular column structure, the soil moisture content is measured by the output frequency of the frequency divider in the probe, and the capacitive soil moisture sensor is calibrated. Matteo Francia et al. proposed an original approach that builds fine-grained 2D and 3D soil moisture profiles by relying on a sensor grid. To create a cost-effective operative solution, they have shown that three sensors properly placed in the soil are sufficient to effectively obtain the soil moisture profile [17]. The farmland soil environment monitoring system developed by Xu Sipu et al. realizes the automated collection and storage of soil environment data; it consists of self-configuring, low-power Zigbee network nodes that enable online and continuous monitoring of the soil environment every 5 min [18]. Limin Yu et al. reviewed the previous research conducted in the past two to three decades on soil moisture sensors and summarized the principles and various applications of commonly used soil moisture sensors [19]. They indicated that soil moisture sensors in the future should be developed to achieve high-precision, low-cost, non-destructive, automated, and highly integrated systems. Di An et al. proposed a digital twin with the AIoLT framework to effectively improve the cumbersome pretreatment process and an expensive analyzer to quantify the total soil carbon content process, achieving a very effective and cost-effective soil carbon content accounting method [20]. Nipuna Chamara et al. provided overview guidelines for the design and development of agricultural IoT crop, soil, and microclimate monitoring systems [21].

As for soil information prediction, Pankaj Pal et al. proposed a single probe imitation of multi-depth capacitive soil moisture sensors for a low-cost and energy-efficient IoT-assisted wireless sensor network farm monitoring infrastructure [22]. Two neural network models—an artificial neural network (ANN) and a bidirectional long short-term memory network (BLSTM)—were proposed and compared to estimate the soil water content at different soil depths. Lea Dujić Rodić et al. studied a smart irrigation ecosystem combined with machine learning to ensure optimal water usage. Their work explored the concept of a low-power, LoRa-based, cost-effective system that senses humidity with a high accuracy using deep learning techniques simply by measuring the signal strength of a given underground beacon device [23]. Sagarika Paul et al. used machine learning techniques such as linear regression, support vector machine regression, PCA, and naïve Bayes to predict the soil moisture 12 to 13 weeks in advance [24]. Hongyu Shi et al. used deep reinforcement learning (DRL) to optimize task scheduling at the edge of the IoT and proposed an EC-AIoT CMS based on DRL optimization [25]. Sonia Naderi et al. proposed a low-cost, reliable wireless soil moisture sensing system to enable efficient spatial–temporal data collection, in which a random forest, a Gaussian process, and a support vector regressor were used to calibrate the system [26]. Arash Heidari et al. proposed a new deep Q learning approach that uses a Markov decision process (MDP) to solve the IoT edge offloading-enabled blockchain problem [27].

Moreover, reference [28] reports the study of monitoring applications based on IoT technologies, big data, and WSNs; some of the applications are also exemplified, including SHM. Articles [29,30,31] conclude with further outlooks on agricultural IoT technologies, suggesting that there is still room for future development, including the virtualization of sensors and IoT devices and increased scalability, heterogeneity, interoperability, and security, to take full advantage of the cloud-based IoT. Safa Mohammed Sali and others used a Raspberry PI and Arduino to drive the Smart Rover military surveillance robot vehicle from a smartphone [32].

At present, research on agricultural planting is continuing to increase, therefore increasing research into the control of agricultural intelligent systems. This situation has led to the need for smart sensors, as the front end of the agricultural IoT, to possess network communication abilities. Sensors with a transmission function are the basis of continuous data acquisition in real time. Although the above systems have achieved the collection and processing of soil information, they do not use the IoT interface technology to obtain more useful information and lack the unified management and independent research and development of the supporting visualization platform, leading to many system maintenance difficulties.

Based on this analysis, an IoT-based soil diagnosis framework is proposed. The framework consists of large-scale sensor nodes and multiple sink nodes, each of which can communicate using IPv6. The framework can be broadly summarized in the following: Firstly, the data are sent from the sensor node, then the data from multiple sensor nodes are aggregated and sent from the sink node to the data collection point. The data can be received and processed by the IoT data management platform or they can be received and processed by the cloud computing platform before being passed on to the IoT data management platform. Finally, the data are transferred to specific applications. The adopted cloud database is NoSQL, which is designed for web, mobile, and IoT applications, where the data are stored permanently [33].

## 2. Materials and Methods

### 2.1. Working Steps

The proposed system is mainly concerned with the application of the soil IoT. This section will detail the approaches to designing the system focusing on soil data flow.

The first step is the data transfer process between the individual sensors and the microprocessor; the sensors used in this research all operate on the principle of using sensitive elements to convert a sensed non-electrical quantity (soil temperature, MC, and pH values) into an electrical quantity through a series of conversion elements. The conversion element is an integrated node board with a PIC18F4620 microprocessor and an antenna module (MRF24J40) developed by Newcastle University. The node board allows the user to use MPLAB software for the autonomous development of the microprocessor on board, and since all sensors output analogue signals, additional 10-bit ADC functions are required to convert them to digital signals.

The second step is inter-cluster communication. Sending data from each node directly to the sink would be too cumbersome for real-world applications; thus, for ease of use, the WSN technique can be applied here by setting one node as the master and the other nodes as slaves.

The third step is to send the data from the cluster head nodes to the sink, which is the Raspberry Pi Zero W microcomputer. Thus, all soil information can be recorded and stored.

The fourth step is to transfer the data from the microcomputer to the cloud platform. This step requires the data-sending method provided by the cloud platform to be written into the microcomputer. At this step, users can view their soil data on the cloud platform. A deep-learning-based prediction mechanism is also in operation here.

**Figure 2:**
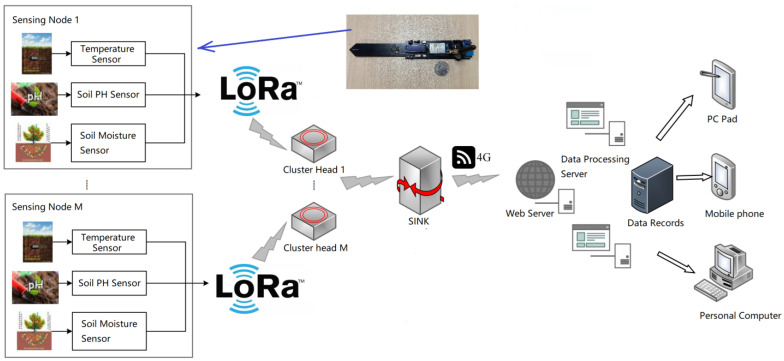
Diagram of the proposed soil monitoring system.

The final step is to download and transfer the data from the cloud to the mobile application. The code for this step was written in Android Studio software. In the final data presentation, the user interface was designed with customizable soil parameters in order to give the user a more intuitive sense of whether the soil is in a good condition; this allows users to visually and easily observe their soil data in real time from the mobile app. Figure 2 shows the whole system structure.

### 2.2. Hardware Implementation and Sensor Selection

In order to implement the practical system, the choice of soil sensors and the formulae for converting the electrical parameters into their relative counterparts are important.

Firstly, there are many different types of temperature sensors which are generally divided into two main categories, namely thermocouple and thermistor temperature sensors [34]. In this research, we used a negative temperature coefficient (NTC) thermistor sensor [35], i.e., a Panasonic electronic temperature sensor, which has been widely used in aerospace engineering due to its high sensitivity, high accuracy, and low cost. The measurement range is from −40 °C to +125 °C. The relationship between temperature and resistance is given below:

(1) $$Temperature=\frac{1}{\frac{B}{\log(\frac{R}{R_{0}})}+\frac{1}{298.15}}-273.15$$

where *R* represents the variable resistance value, *R*_0_ = 100,000, and *B* is the value of the thermistor (*B* = 4725).

There are two common types of soil moisture sensors, namely resistive and capacitive moisture sensors [36]. The resistive soil moisture sensor used in this study consists of a number of small cells formed by two electrodes passing through a moisture sensing layer, which causes a change in the resistance as the number of small cells changes. To verify the accuracy of the moisture sensor measurements, we calibrated the true value of the MC with a commercial device—the KT-80 dual-function moisture meter from Klortner Technologies, Italy—which has an overall accuracy of more than 97%.

For the calculation of the moisture, a simple linear model, y = *a*x + *b*, is used. Moisture = *a* × *ADCresult* × 3.3/1023 + *b*, where *a* and *b* are constants and *ADCresult* × 3.3/1023 is the sensor’s resistance value. At first, the node board only outputs *ADCresult* × 3.3/1023. When the moisture sensor is placed in a dry place, the moisture should be equal to zero and the output should be zero. When the moisture sensor is placed in a cup full of water, the moisture should be equal to one hundred percent. The sensor output was almost equal to 0.845. We can obtain a system of quadratic equations based on these data:

(2) $$\{\frac{0=a\times 0+b}{100=a\times 0.845+b}$$

and thus we can obtain the calibration relationship between moisture and resistance for this type of sensor as

(3) Moisture=37×ADCresult×3.3/1023

Raspberry Pi Zero W is an updated version of Raspberry Pi Zero and the configurations of both are almost identical [37]. It is an ARM-based microcomputer mainboard with a microSD card as the memory drive, a USB data port, and a USB power port around the card mainboard to connect a keyboard and a network cable. The microcomputer can be programmed in python for networking and then programmed to upload and receive functions. In this work, it is necessary to receive and send data using a wireless transmission module.

**Figure 3:**
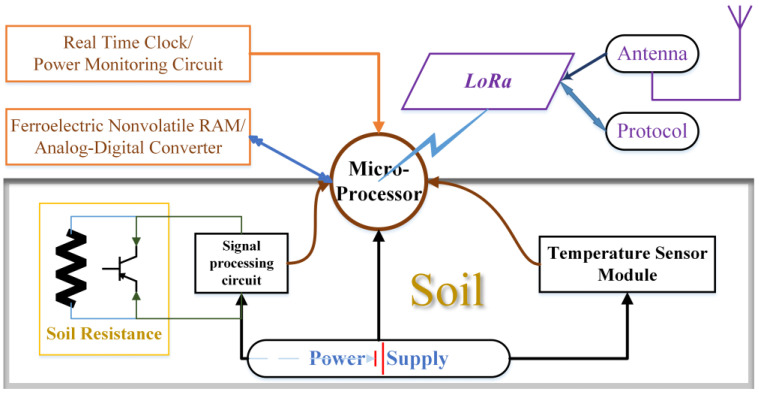
Structural diagram of the proposed soil wireless node.

In terms of the choice of cloud platform, the Thingspeak cloud platform is chosen. As it provides an API key for reading data from the channel in json format, it is also necessary to download the JSON library to the mobile phone’s Android Studio, and the OkGo library is also needed to design the reading function of the mobile application in order to design the network encapsulation and other functions in a more flexible way [38]. Figure 3 shows the wireless node diagram.

### 2.3. Combined Prediction Model of Moisture and Temperature Based on Deep Reinforcement Learning

A deep Q network (DQN) is a classical algorithm for deep reinforcement learning which makes improvements to the problems of reinforcement learning. The improvements to the DQN based on the incorporation of neural networks include the following two main aspects [39]:

(1) An experience replay mechanism is used, which constructs a pool of experience. The smart body stores sample data in the experience pool after each execution of an action. A random sample is taken from the experience pool at each training session to clear the correlation of the observation sequence.
(2) The DQN uses a current value network and a target value network. The target value network is used to assist in the calculation of *Q* values, and the current value network is updated to the target value network at regular intervals.

The network parameters θ are updated using time difference deviation δ with the loss function L(θ). The target *Q* value (*Q_target_*) is calculated as:

(4) $$Q_{\mathrm{target}}=r+\gamma \underset{a^{\prime }}{\max}Q(s\prime ,a\prime ,\theta^{-})$$

where *r* is the immediate reward for performing actions, γ is the discount factor, s′ is the state in the next time slot, a′ is the action in the next time slot, and $\theta^{-}$ is the target network parameter. δ is calculated as:

(5) $$\delta =r+\underset{a\prime }{\max Q(s\prime ,a\prime ,\theta^{-})}-Q(s\prime ,a\prime ,\theta^{-})$$

**Figure 4:**
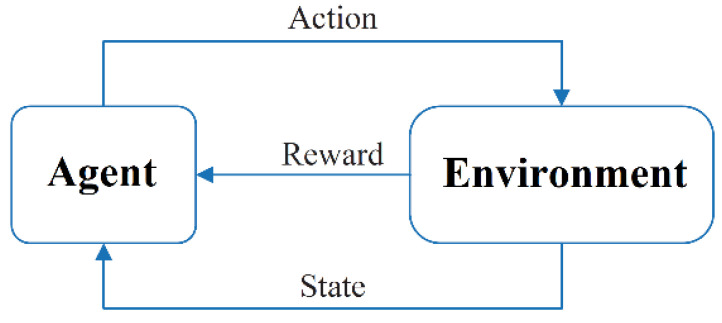
Block diagram of the reinforcement learning system.

$L(\theta )=\delta^{2}$. Figure 4 presents a diagram of reinforcement learning.

**Figure 5:**
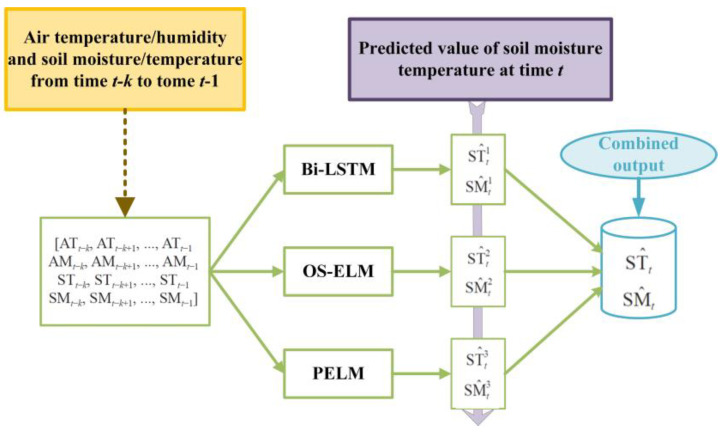
Structure diagram of the combination forecasting model.

Here, the individual models in the combined model are called base models, and the base models used in the combination are Bi-LSTM [40], OS-ELM, and PELM [41]. These three single models have their own advantages and disadvantages. In order to maximize the prediction performance of the single models and improve the accuracy of soil MC and temperature prediction, a combined DQN-Bi-OS-P prediction model based on deep reinforcement learning is investigated. The structure of the combined prediction model is shown in Figure 4. The inputs to the combined model are air temperature, humidity, soil moisture, and temperature data from time *t*−k to time *t*−1, which are mapped to different Bi-LSTM, OS-ELM, and PELM models, where the predicted values of the output of each model are $\left[S{\hat{T}}_{t}^{1},S{\hat{M}}_{t}^{1}\right]$, $\left[S{\hat{T}}_{t}^{2},S{\hat{M}}_{t}^{2}\right]$, and $\left[S{\hat{T}}_{t}^{3},S{\hat{M}}_{t}^{3}\right]$. The predictions from each model are combined to obtain the final prediction value, as shown in Figure 5.

In order to improve the combined prediction results of the Bi-LSTM, OS-ELM, and PELM models, a DQN deep reinforcement learning algorithm was introduced for weighted summation to obtain optimized prediction values. Based on the Markov decision process, the state space ***S***, action space ***A***, and reward function ***R*** are modeled as follows:

(1) State space ***S***. The state space matrix contains the prediction result weights for each base model as shown in Equation (6):

(6) $$S=\left[\omega_{1},\omega_{2},\omega_{3}\right]$$

where $\omega_{1}$, $\omega_{2}$, and $\omega_{3}$ are the prediction result weights of the three base models. The initial ***S***_0_ is set as [1/3, 1/3, 1/3].

(2) Action space ***A***. The action space matrix contains the actions that increase and decrease the prediction weights of each base model, as shown in Equation (7):

(7) $$A=[\begin{array}{cc} +\Delta w & -\Delta w\\ +\Delta w & -\Delta w\\ +\Delta w & -\Delta w \end{array}]$$

where Δw indicates the amount of weight to be added or subtracted for each action performed.

(3) Reward function ***R***. The setting of the reward function is an important issue in deep reinforcement learning, and the immediate reward function ***R*** obtained after each action is set as shown in Equations (8) and (9).

(8) $$MAE(T)=\sum_{t=1}^{N}\left|w_{1}^{T}{\hat{y}}_{t}^{1}+w_{2}^{T}{\hat{y}}_{t}^{2}+w_{3}^{T}{\hat{y}}_{t}^{3}-y_{t}\right|/N$$

(9) $$R=\{\begin{array}{c} k+MAE(T)-MAE(T+1),ifMAE(T+1)<MAE(T)\\ -k+MAE(T)-MAE(T+1),ifMAE(T+1)\geq MAE(T) \end{array}$$

where *T* denotes the *T*th action, $w_{1}^{T},w_{2}^{T},w_{3}^{T}$ are the three base models’ prediction weights after the *T*th action, respectively, ${\hat{y}}_{t}^{1}{\hat{y}}_{t}^{2}{\hat{y}}_{t}^{3}$ denote the predicted values of the combined model, *y_t_* is the actual value, and *N* denotes the number of samples in the training set. After executing the action, if the *MAE* of the combined model predictor is less than the previous result, a reward will be given. An additional bonus with a fixed value of *k* is set when *MAE*(*T* + 1) < *MAE*(*T*) to avoid the problem of sparse rewards when the *MAE* enhancement is too small.

The DQN-Bi-OS-P prediction model adopts the ε-greedy exploration strategy [42]. Eventually, when the training times are satisfied, the optimal strategy and the optimized weight matrix are output $\left[\omega_{1},\omega_{2},\omega_{3}\right]$.

### 2.4. Field Soil Data Acquisition

**Figure 6:**
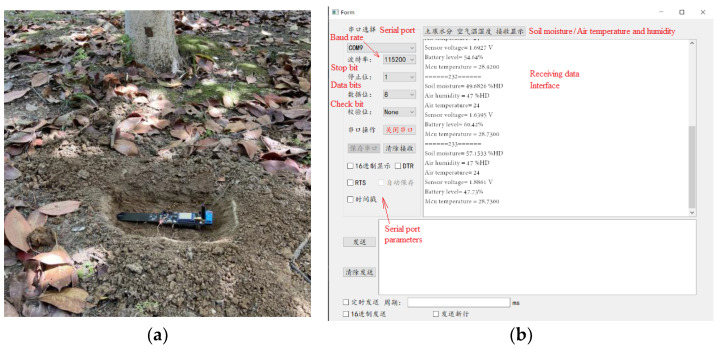
Field data acquisition: (**a**) field testing of a soil wireless sensor node; (**b**) data-receiving interface on a PC terminal.

The proposed system consists of field terminal nodes and a cloud-based data management platform. The field terminal nodes measure the values of the monitored parameters and send them to the cloud data management platform through a 4G data channel. The cloud data management platform combines the server MySQL database to realize the management functions of the soil data interface, data storage, and data interaction. The field terminal node installation and its testing are shown in Figure 6.

After the data acquisition system was built, the terminal nodes were deployed in the test bases for long-term data acquisition trials. The test sites were located in Xuan Wu District, Nanjing City, Jiangsu Province, China. Xuan Wu District has a warm–temperate maritime monsoon climate, is close to the eastern coast, and is relatively humid, with an average annual precipitation of about 900 mL. Loamy soil from the Nanjing Zi Jin Shan Mountain was selected as the test soil.

The soil MC nodes were placed about one meter away around the crop roots, and they were buried in the soil with flags in the ground. In this work, four underground nodes with depths of 10, 20, 30, and 40 cm were used for testing. The entire test period was from 4 January 2022 to 5 January 2023.

### 2.5. Mobile Experimental Platform

The data displayed on the phone are temperature and humidity values. The LoRa module transmits data every 15 min, and the data measured by the system are basically the same as the parameters displayed by the calibration equipment. Similarly, the information on the mobile phone is refreshed every 15 min, and the data displayed on the mobile phone are accurate to two decimal places, ensuring the accuracy and real-time monitoring of the data.

**Figure 7:**
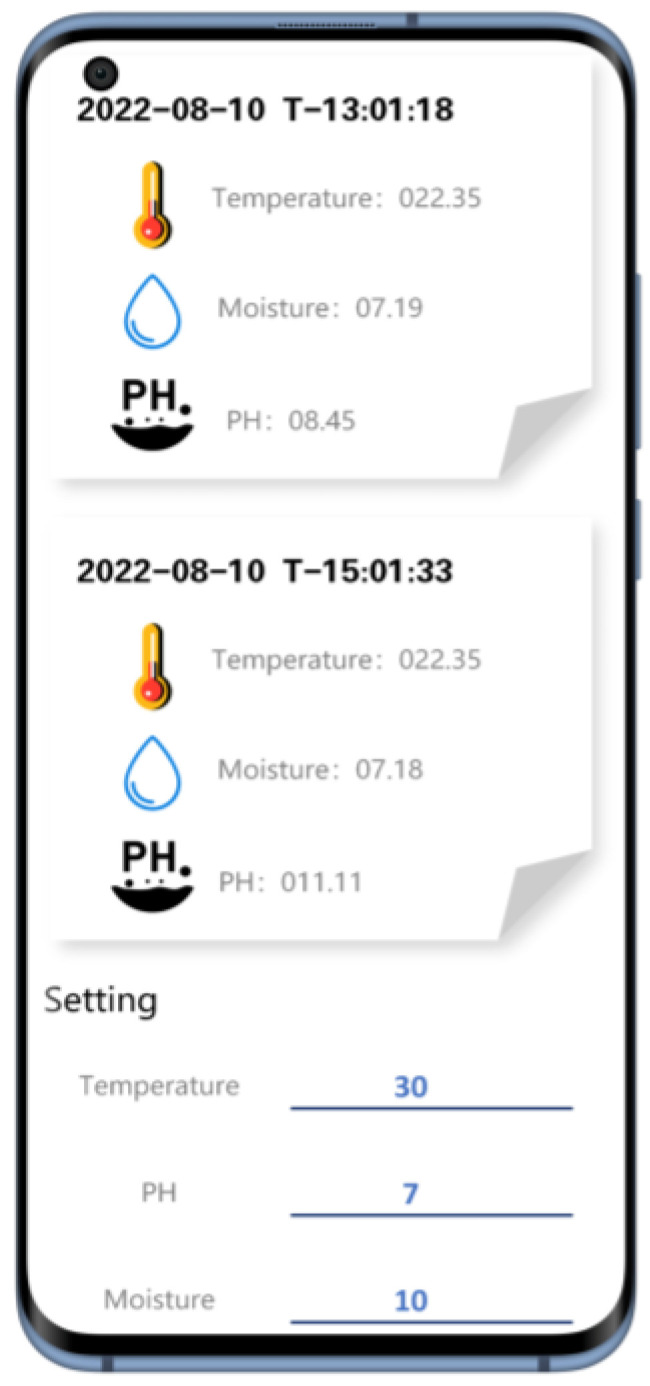
Testing data interface for visualization.

Figure 7 is a test screenshot of the data interface.

### 2.6. Experiments on Multilayer Seasonal Variation in Soil Temperature

**Figure 8:**
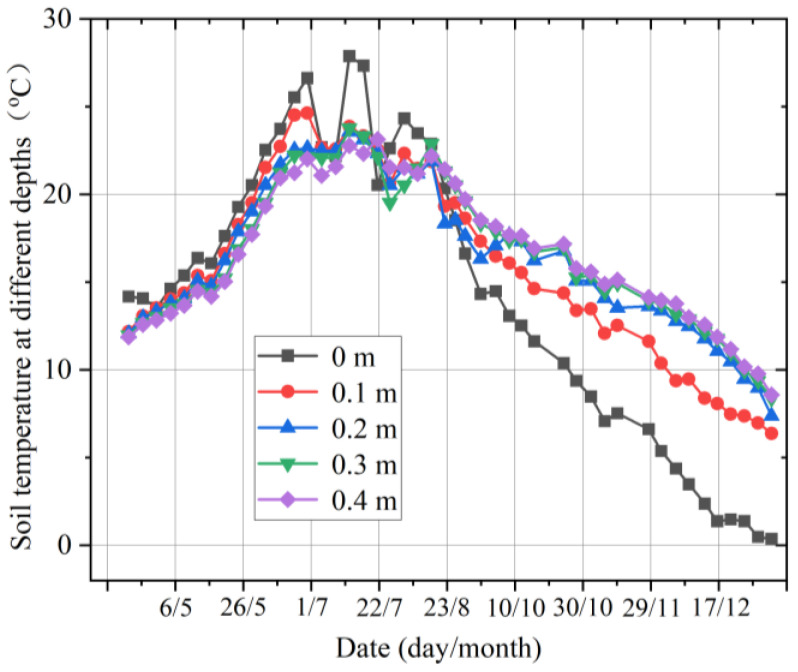
The annual temperature changes in the soil at different depths.

Long-term monitoring was conducted in the National Forest Park of Zi Jin Shan, Nanjing, China. The temperature and MC data were collected at five different depths. After 12 months of operation, it can be observed that there is a very clear annual variation in the soil temperature, with the temperature at the same depth generally rising and then falling, similar to the first half of a sinusoidal variation. The shallower the soil depth, the greater the magnitude of the temperature change. There is a certain temperature difference between soils at different depths, which varies with the seasons, weather, and other factors (Figure 8).

There is a more pronounced seasonal pattern in the forest soil temperature in the different soil layers. Generally speaking, it is more scientific to divide the seasons into the meteorological seasons, which are Spring in March, April, and May; Summer in June, July, and August; Autumn in September, October, and November; and Winter in December, January, and February. In the following, the seasonal patterns in soil-temperature changes in the forest’s multiple soil layers will be analyzed in the order of spring, summer, autumn, and winter.

**Figure 9:**
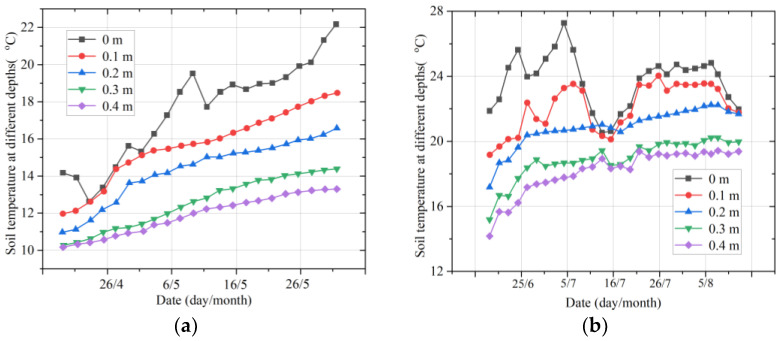

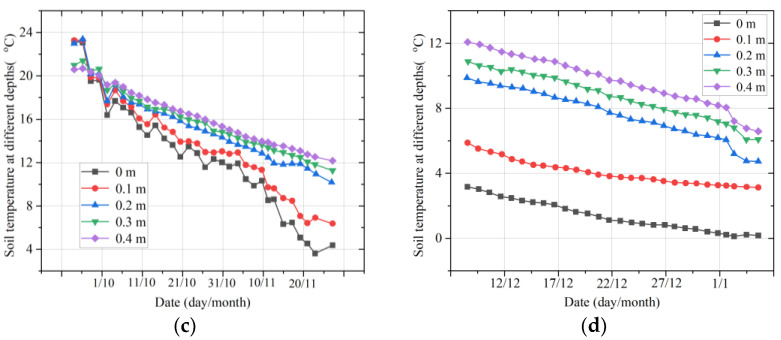
The temperature changes in the multiple soil layers in different seasons: (**a**) temperature variation in spring; (**b**) temperature variation in summer; (**c**) temperature variation in autumn; (**d**) temperature variation in winter.

As can be seen from Figure 9, there is a clear temperature difference between the multiple soil layers. In spring, the soil temperature tends to increase broadly at different depths as the temperature warms up, among other factors. The magnitude of the temperature variation is greater in the summer for the multilayered soil. The overall trend is a decrease in temperature values with increasing depth. In middle and early September, the temperature near the ground varies considerably, with the temperatures at 0.1, 0.2, and 0.3 m being relatively stable, and the temperatures at 0.1 m being close to those at 0.2 m and higher than those at 0 m (near the ground layer). From the beginning of October, the temperature gradually increases with the depth of the soil layer. In winter, the temperature of the soil layers at all depths showed a general downward trend, indicating that as the winter temperature became colder, the temperature of the soil layers also decreased. The rate of the temperature decrease at all depths of the soil decreased as January progressed, and as the depth of the soil increased, the soil temperature increased.

**Figure 10:**
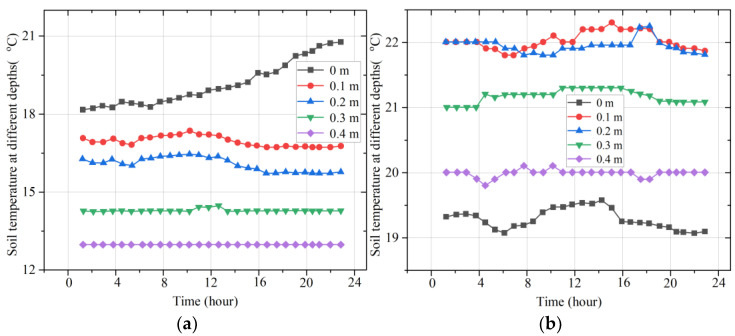
The temperature changes in the multiple soil layers on two days: (**a**) temperature on 25 May; (**b**) temperature on 23 October.

Moreover, typical representative days of the weather in different seasons were selected to analyze the variation in multilayer soil temperatures in one day in order to summarize the daily variation patterns in multilayer soil temperatures in different seasons, as shown in Figure 10.

The observations show that the near-ground soil temperature tends to rise slowly in clear weather (25 May), indicating that heat can accumulate in the near-ground layer. As the depth of the soil layer increases, the temperature decreases and the magnitude of the temperature change becomes smaller. The other example is the daily variation in multilayer soil temperatures on 23 October, a representative day in autumn. The maximum temperature on that day was 27 °C, the minimum temperature was 19 °C, the rainfall was 4 mm, and the weather was foggy with thundershowers. The observations show that, due to the rainfall, the near-ground soil had less opportunity to accumulate heat and the near-ground temperature varied more smoothly. The soil temperature varied less at other depths, with a variation interval of less than 1 °C.

According to these findings, the soil moistures in different layers also exhibit different variation rules in different seasons. This section will demonstrate and analyze the seasonal variation in soil moisture in the different layers.

**Figure 11:**
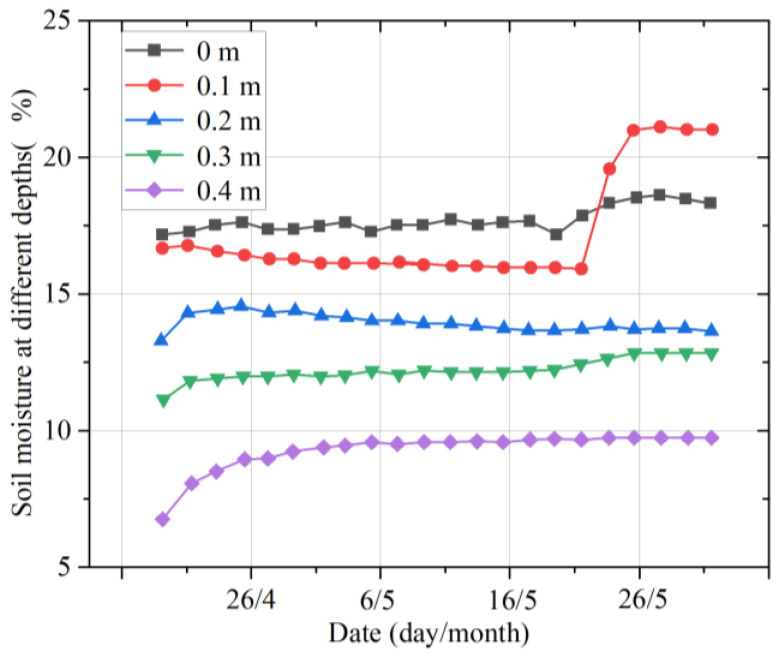
Moisture changes in multilayer soil in spring.

Figure 11 shows the change in the soil moisture in the different layers in spring. It can be observed that the change in soil moisture at different depths in spring is relatively stable. In mid-early May, the soil moisture at different depths decreased in the order 0 m > 0.1 m > 0.3 m > 0.2 m > 0.4 m. However, at 0.1 m, the soil moisture significantly increased in late May, and the order of soil moistures changed to 0.1 m > 0 m > 0.3 m > 0.2 m > 0.4 m. At depths of 0.2 m and 0.3 m, the soil moistures were similar.

**Figure 12:**
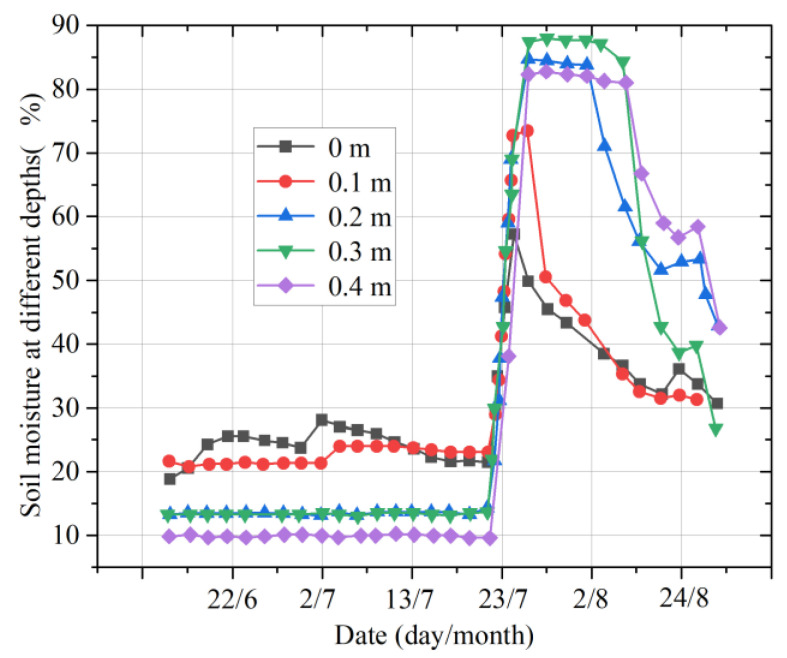
Moisture changes in multilayer soil in summer.

Figure 12 shows the variation curves of soil moisture in the different layers in summer. From the beginning of June to the beginning of July, the changes in soil moisture at different depths were relatively stable. The soil moisture at a depth of 0.2 m was almost the same as that at 0.3 m. In late July, because of the frequent and strong rainfall, the soil moisture at different depths increased sharply. On 21 July, the soil moisture reached the maximum at all depths, and the maximum value was 88.19% at 0.3 m.

**Figure 13:**
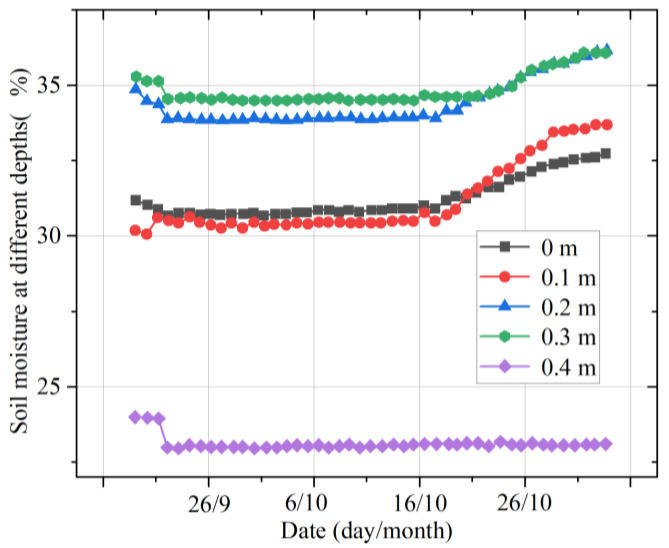
Moisture changes in the multilayer soil in autumn.

Figure 13 shows the change in the multilayer soil moisture in autumn. It was found that the changes in soil moisture at different depths were relatively stable. Before 18 October, the order of soil moisture according to depth was 0.3 m > 0.2 m > 0 m > 0.1 m > 0.4 m. After 18 October, the humidity at a depth of 0.2 m began to approach that at a depth of 0.3 m, with a small gap between the two and a clear rising trend. At the same time, the humidity at a depth of 0.1 m increased gradually, exceeding that at 0 m. The humidities at depths of 0.1 and 0 m both rose, but that at a depth of 0.1 m grew at a faster rate.

**Figure 14:**
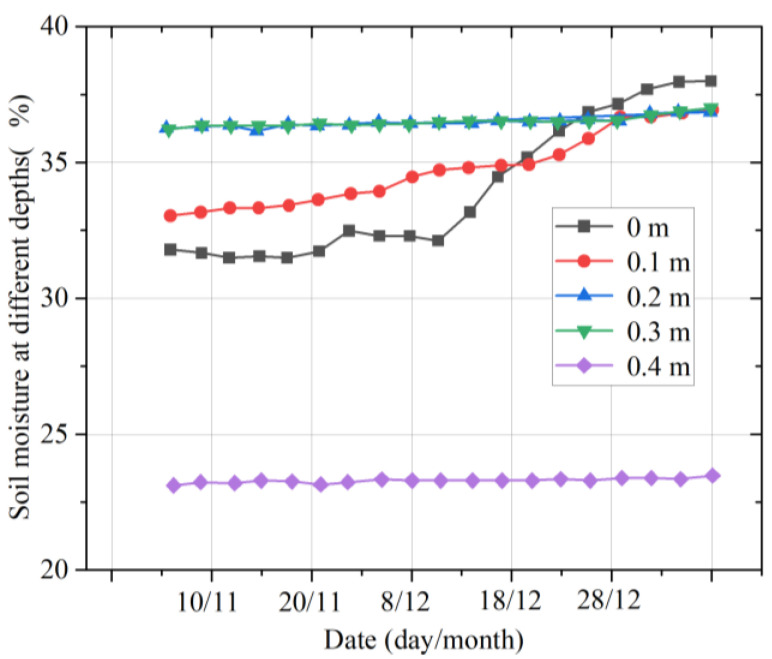
Moisture changes in multilayer soil in winter.

Figure 14 shows the variation in the multilayer soil moisture in winter. The observations show that at depths of 0.2, 0.3, and 0.4 m, the soil moisture remains roughly the same, and at 0.2 m and 0.3 m, the soil moistures are nearly equal. At 0 m (near the ground surface) and 0.1 m deep, the soil moisture steadily rises as winter progresses, and after 26 December, the soil moistures at 0.1 m and 0.2 m are very close to that at 0.3 m, at about 37%.

Environmental factors such as sun, rain, snow, haze, and other weather affect the ground humidity, which then affect the multilayer soil humidity. Humidity also has a certain influence on the temperature at this depth, so it is of clear significance to study the influence of the weather on the soil moisture in multiple layers. Two representative weather conditions are selected to investigate the influence of the weather on the multilayer soil moisture.

## 3. Results

**Figure 15:**
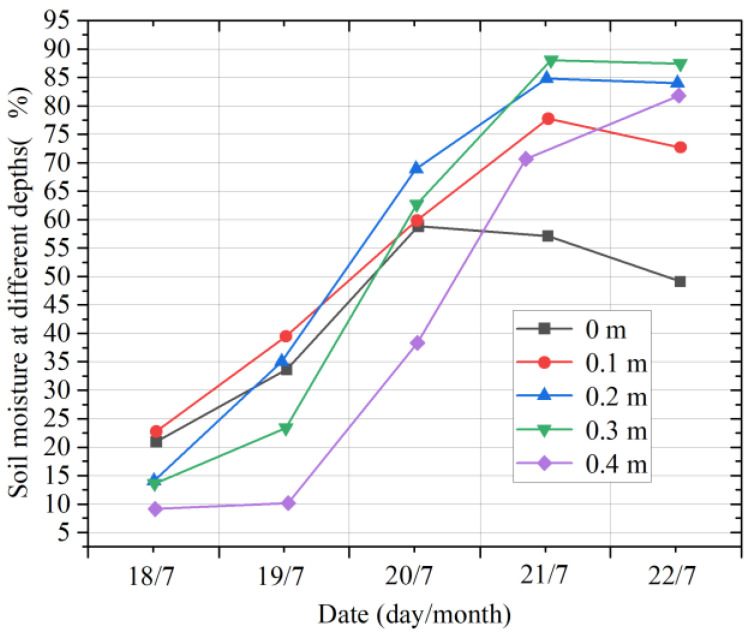
Moisture changes in multilayer soil on rainy days.

Figure 15 shows the change in soil moisture in the different layers from 18 July 2022 to 22 July 2022. It can be observed that continuous rainfall leads to a steep increase in the soil moisture in each layer. With a decrease in the rainfall and an increase in the air temperature, the growth in the multilayer soil moisture slows down, and the moisture tends to decline. Continuous rainfall increases the moisture in the air, which reduces the evaporation of water from the topsoil; thus, the soil moisture increases sharply at all levels. With the increase in light, air temperature, wind speed, and other environmental factors, the evapotranspiration of soil water will increase, and thus the growth rate of multilayer soil moisture will slow down and may even decline.

**Figure 16:**
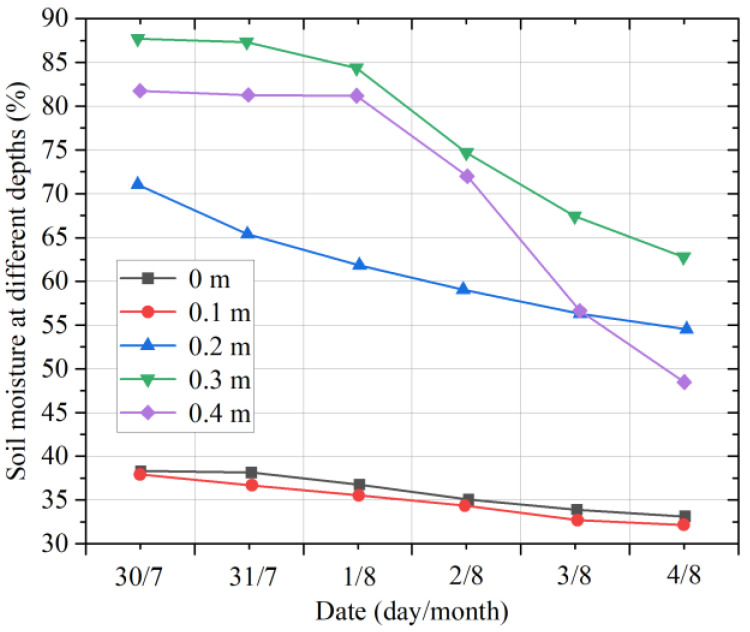
Moisture changes in multilayer soil on sunny days.

Figure 16 shows the changes in the soil moisture in different layers from 30 July 2022 to 4 August 2022. It can be found that continuous sunny days lead to a decrease in the soil moisture in each layer. Due to the slow infiltration rate of rainwater and the loss of rainwater in the process of infiltration, the soil moisture at 0.3 m is the highest, followed by that at 0.4 m. Due to the large proportion of sand and gravel in the soil at a depth of 0.4 m, the water storage capacity of the soil at this depth is reduced compared to the upper soil. Therefore, the rate of decline in the soil moisture at the depth of 0.4 m is faster, and the soil moisture at this depth is thus lower than that at a depth of 0.2 m.

The data collected by the data acquisition system from 2022 to 2023 were used as the dataset, including the air temperature, air humidity, soil temperature, soil moisture, and other ecological data relevant to plant cultivation. A total of 90% of the data were used as the training set and 10% as the test set. The training set was used to train the Bi-LSTM, OS-ELM, PELM, and DQN-Bi-OS-P combination models, and then the test set was used to compare and analyze the prediction results of each model.

The input time step for the deep learning prediction model was five, and there were four input dimensions. The dimensions were air temperature, air humidity, soil temperature, and soil moisture. The model was trained using a Windows 10 64-bit operating system, an Intel Core i7-12700H CPU at 2.30 GHz, 16 GB RAM, the Python 3.6 programming language, and a Tensor flow 2.1 deep learning framework.

The Bi-LSTM, OS-ELM, and PELM models were constructed with two network layers of 32 and 16 neurons, respectively, using Tanh as the activation function, Huber loss as the loss function, and Adam as the optimization algorithm. The number of iterations was set to 100. The DQN neural network model in DQN-Bi-OS-P uses two fully connected neural networks with 128 and 64 neurons, respectively. The training hyperparameters were set to a learning rate of 10, a reward discount factor of 0.9, a weight change step of 10-4, an experience pool capacity of 3200, a minimum update batch of 32, a target value network update step of 200, a Huber loss hyperparameter of 1, a total number of training tasks of 10,000, and an exploration probability of 0.1. The Adam optimizer and the ReLU activation function were used.

**Figure 17:**
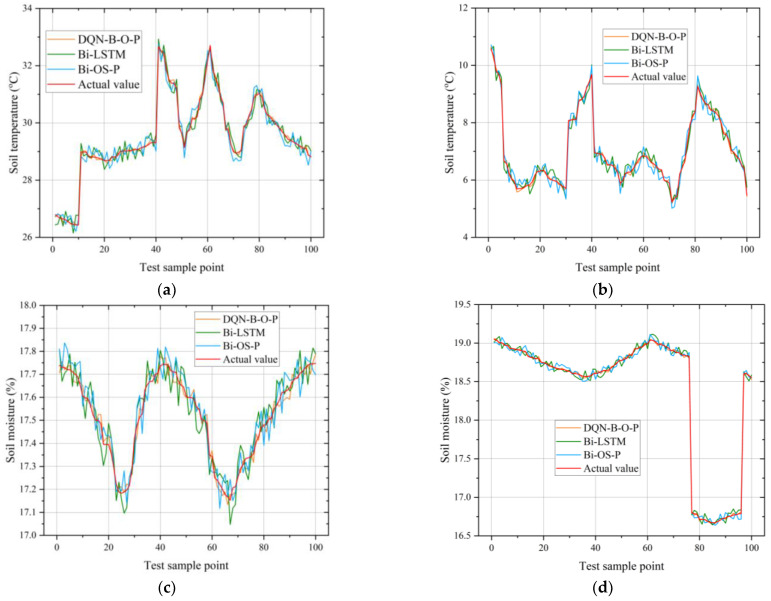
Comparisons of DQN-Bi-OS-P and other models for soil MC and temperature prediction: (**a**) prediction and true values for soil temperature on 5–7 July; (**b**) prediction and true values for soil temperature on 19–21 December; (**c**) prediction and true values for soil MC on 7–9 April; (**d**) prediction and true values for soil MC on 23–25 September.

In the following analysis of the experimental results, the Bi-LSTM, which was the best performing base model, was introduced for a comparison with the other two combined models, and sample points from the four seasons at 20-cm underground were selected for the fitted curves as an example. In Figure 17, it can be seen that the fitted curve of the DQN-Bi-OS-P model is the closest to the true value.

The DQN-Bi-OS-P model was trained to give optimized prediction weights *ω*_1_, *ω*_2_, and *ω*_3_ of 0.330777, 0.331261, and 0.332552 for the tillage soil, respectively. In order to facilitate a comparative analysis of the effectiveness of the DQN-L-G-B model optimization, a weighted average Bi-OS-P model with base model weights *ω*_1_, *ω*_2_, and *ω*_3_ of one-third was introduced into the analysis of the model results. A comparison of the performance of the Bi-LSTM, OS-ELM, PELM, Bi-OS-P, and DQN-Bi-OS-P models using the RMSE, MAE, MAPE, and R2 evaluation indicators regarding the soil tillage MC and temperature test set is shown in Table 1 and Table 2. It is clear that the DQN-Bi-OS-P outperformed the other models without the DQN framework in soil data prediction.

## 4. Discussion

**Figure 18:**
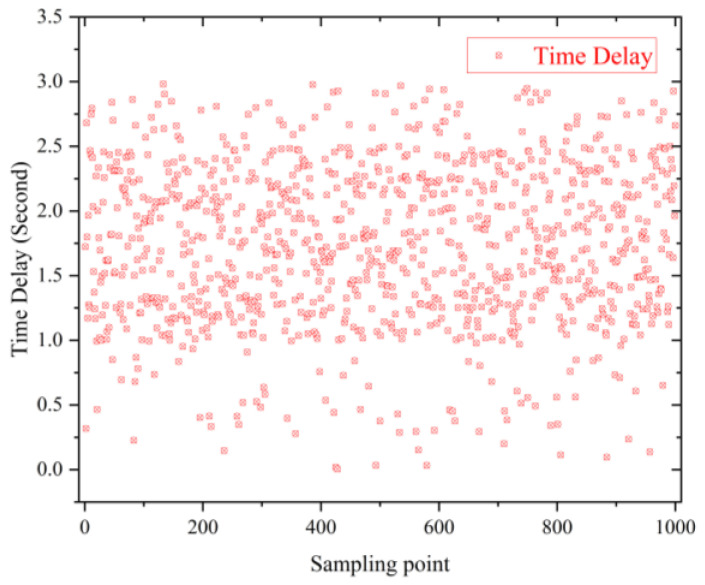
The time-delay result of part data by random selection.

(1) Communication performance of the wireless nodes: The wireless nodes operating at 2.4 GHz based on the IEEE 802.15.4 protocol and deployed underground will suffer from signal attenuation between the nodes and the sink due to the soil properties. Message loss is inevitable when there is heavy rain or snow. Hence, we gathered statistics on the time delay between the data-receiving moment at the mobile phone with the data-collection moment in the preset schedule, as shown in Figure 18. We found that none of the delays were longer than 3 s, and most of them were distributed between 1.0 and 2.5 s. The main reason for this is the high performance of the Raspberry Pi, which connects with the cloud efficiently.

(2) Power consumption of the wireless node: The underground node uses an LS33600 SAFT battery as a power source, which can last for nearly 6 months. The majority of energy consumption is from wireless transmission. Here, we adopted the conventional sleep mode for the micro-controller unit; in addition, a real-time clock chip takes charge of the awakening function in a specified time period. The work/sleep duty cycle is nearly 10~12%, which represents a significant reduction compared to the original value.
(3) Failure process: In terms of node failures, if the non-response problem occurs, a worker can be notified via the designed mobile phone app, regardless of the size of the farm. We also used a heartbeat packet to detect the network performance, which includes routing sequence information; hence, we could locate the failed node as soon as possible.
(4) The LoRa module SX1278 has a maximum transmitting power of 100 mW. The power consumption of the AD7356 analog-to-digital converter and the OPA627 preamplifier chip is about 111 mW. Moreover, the soil sensors have two modes: working and sleeping. Their duty cycle is usually set at about 3% and, considering that the node only needs to collect data every 4 h, the power consumption can be calculated at about 3000 mW per day. When using a 3.7 V 30 Ah lithium battery pack, one WASN node can normally operate for 37 days. Therefore, the designed WASN basically meets the application requirements for agricultural soil monitoring.

## 5. Conclusions

In this work, firstly, a soil quality monitoring system for farms was successfully designed and implemented, allowing users to check the soil condition of their farmland at any time via their mobile phone and to set soil parameter thresholds in the mobile app. The system sends detected soil data to the user at 15 min intervals, which ensures reliability and allows the user to easily determine the state of their soil by changes in soil temperature and humidity regardless of the time or their location. Next, the DQN-Bi-OS-P combined model was investigated based on deep reinforcement learning methods to predict the soil tillage moisture and temperature. The field experimental results show that the combined model can predict the soil moisture and temperature more accurately using soil data from multiple soil layers. The created system can help people to quickly assess the state of the soil; for example, the system can be used to routinely check whether the soil quality of a piece of land has changed significantly. The work also demonstrated that mobile apps can be effectively applied in IoT-based wireless multiple sensing monitoring. However, there are limitations to this study. We have not yet tested the stability of the entire monitoring system under extreme conditions, such as saline–alkali land, strong winds, heavy rain, and thunderstorms, which will have a significant impact on the final prediction results.

With people’s increasing attention to health issues, wearable medical monitoring devices have seen significant developments in recent years. Flexible sensor front ends, as an important part of these wearable devices, have also received increased attention. Compared to existing health monitoring devices on the market, which have disadvantages such as large sizes, poor wearability, and high prices, we believe that the flexible materials can not only be applied in precision agricultural monitoring, but also have great potential in wearable technology. At present, the main flexible materials exhibiting remarkable progress include ion gel materials [43], inorganic nanomaterials [44], particle composite materials [45], etc. These physiological parameters, such as the human body temperature, skin moisture, and even ECG signals, can be displayed visually in real time through the smart phone app designed in this paper [46]. Of course, in the follow-up application of flexible sensor platforms, there will also be errors caused by electromechanical coupling and material differences, which will be a focus of our future research.

In future research, unmanned aerial vehicles could be adopted as the moving sink. This could strongly enhance the reliability and stability of the wireless communication, including cross-media communication, i.e., communication through soil and air. Meanwhile, further data analyses, including edge computing and the tomography of roots and soils, should also be introduced and investigated for different critical safety applications. Due to their high flexibility and compliance, flexible electronic devices can realize a natural, non-invasive interaction between electronic devices and the human body; thus, we will further study how to apply the information monitoring devices in this paper to wearable technology in future work.

## Figures and Tables

**Table 1:** Results of various models for soil temperature prediction.

Model	RMSE	MAE	MAPE	*R^2^*	Iteration/s
PELM	0.757	0.505	0.0199	0.915	28.41
OS-ELM	0.765	0.533	0.0208	0.914	25.40
Bi-LSTM	0.737	0.502	0.0183	0.916	18.67
Bi-OS-P	0.729	0.488	0.0181	0.927	19.35
DQN-Bi-OS-P	0.699	0.454	0.0166	0.932	9.56

**Table 2:** Results of various models for soil moisture prediction.

Model	RMSE	MAE	MAPE	*R^2^*	Iteration/s
PELM	0.471	0.133	0.0397	0.994	25.77
OS-ELM	0.470	0.147	0.0468	0.994	22.38
Bi-LSTM	0.463	0.112	0.0251	0.995	15.56
Bi-OS-P	0.454	0.105	0.0313	0.995	15.49
DQN-Bi-OS-P	0.431	0.091	0.0215	0.996	6.3

## Data Availability

Not applicable.
